# Bismuth Modified Carbon-Based Electrodes for the Determination of Selected Neonicotinoid Insecticides

**DOI:** 10.3390/molecules16064451

**Published:** 2011-05-27

**Authors:** Valéria Guzsvány, Zsigmond Papp, Jasmina Zbiljić, Olga Vajdle, Marko Rodić

**Affiliations:** Department of Chemistry, Biochemistry and Environmental Protection, Faculty of Sciences, University of Novi Sad, Trg D. Obradovića 3, 21000 Novi Sad, Serbia; Email: zsigmond.papp@dh.uns.ac.rs (Z.P.); jasmina_zbiljic@yahoo.com (J.Z.); goga03@neobee.net (O.V.); rodic.marko@gmail.com (M.R.)

**Keywords:** bismuth modified electrodes, voltammetry, neonicotinoids, clothianidin, imidacloprid

## Abstract

Two types of bismuth modified electrodes, a bismuth-film modified glassy carbon (BiF-GCE) and a bismuth bulk modified carbon paste, were applied for the determination of selected nitroguanidine neonicotinoid insecticides. The method based on an *ex situ* prepared BiF-GCE operated in the differential pulse voltammetric (DPV) mode was applied to determine clothianidin in the concentration range from 2.5 to 23 μg cm^−3^ with a relative standard deviation (RSD) not exceeding 1.5%. The tricresyl phosphate-based carbon paste electrodes (TCP-CPEs), bulk modified with 5 and 20 w/w% of bismuth, showed a different analytical performance in the determination of imidacloprid, regarding the peak shape, potential window, and noise level. The TCP-CPE with 5% Bi was advantageous, and the developed DPV method based on it allowed the determination in the concentration range from 1.7 to 60 μg cm^−3^ with an RSD of 2.4%. To get a deeper insight into the morphology of the bismuth-based sensor surfaces, scanning electron microscopic measurements were performed of both the surface film and the bulk modified electrodes.

## 1. Introduction

In the search for alternative environment-friendly electrode materials, the recently introduced *in situ* and *ex situ* prepared bismuth-film electrodes (BiFEs) have been shown to offer performance comparable to traditionally used mercury-film electrodes. Nowadays, in numerous electroanalytical laboratories many types of BiFEs have been proven to be applicable for different target analytes, first of all trace metals [1,2,3,4,5,6]. On the other hand, electrochemical reduction of organic compounds and their determination have been explored to a much lower extent, and there are only a small number of papers dealing with the analysis of some different pesticides [7,8,9,10,11,12], dyes [13] and drugs [14,15].

Bulk modification of different carbon-based electrodes with elementary bismuth has been studied much less than surface modification with the same element. Among the various electrode materials available for the bulk modification with bismuth powder, carbon pastes have attracted significant attention due to their inherent properties, such as simple preparation, fast and simple surface renewal and also ease of modification [16]. Bismuth modified carbon paste electrodes were successfully applied for the determination of selected metal ions [16] and cysteine [17].

**Figure 1:**
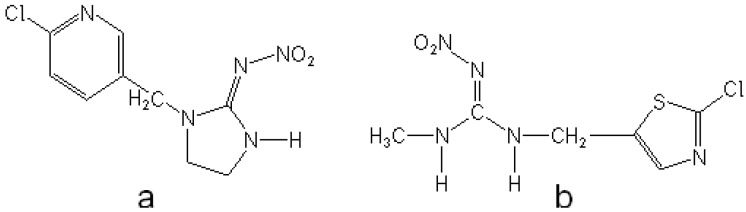
Selected members from two different generations of the neonicotinoid insecticides: imidacloprid (**a**) and clothianidin (**b**).

In the last several years, neonicotinoids (Figure 1) have represented one of the most important classes of insecticides on the market. As potent agonists, they act on the nicotinic acetylcholine receptors of insects [18,19,20]. Their widespread use however, results in their frequent appearance in the environment and foods. Because of that, there is a growing need for the development of fast and simple methods for the characterization and determination of neonicotinoids. These insecticides are mainly determined by HPLC with diode array [21,22,23], mass-spectrometric [24,25], thermal lens spectroscopic [26] and amperometric [27] detection. Several papers have also appeared dealing with the voltammetric determination of neonicotinoids in model systems and different real samples [28,29,30,31,32,33,34,35].

The objective of the present work was to develop voltammetric methods for the determination of two nitroguanidine neonicotinoid insecticides, clothianidin and imidacloprid, using bismuth modified carbon-based electrodes (bismuth-film modified glassy carbon electrode, BiF-GCE and bismuth-powder modified carbon paste electrodes).

## 2. Results and Discussion

### 2.1. Bismuth-Film Modified Glassy Carbon Electrode for the Determination of Clothianidin

Voltammetric sensing of an analyte depends mainly on the surface characteristics of the electrode, investigated compound and nature of the supporting electrolyte. The quality of an electrode is reflected in the reversibility of various faradaic transformations involving the electron transfer, in the pathways of catalytically and kinetically driven reactions or in specific non-electrolytic interactions such as adsorption [36]. Previous microscopic and spectroelectrochemical studies showed that bismuth can be deposited with a very large variability of structure and compactness of the deposited layer, depending on the plating potential, composition of the plating solution, and nature of the substrate electrode [36,37,38,39,40,41,42].

**Figure 2:**
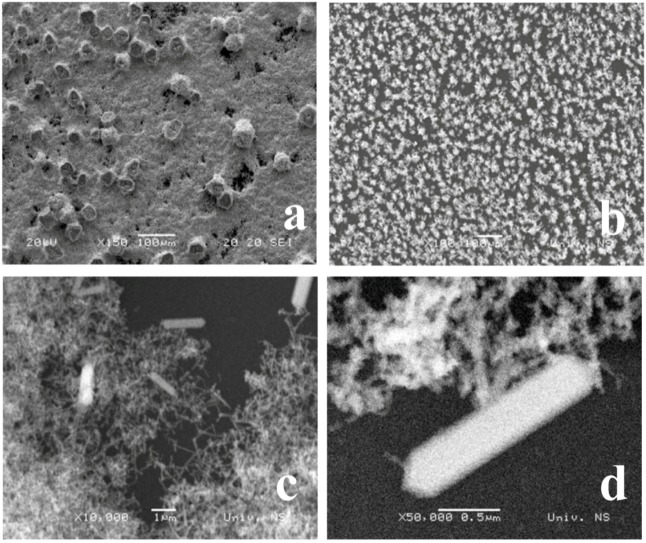
Scanning electron micrographs of bismuth-film deposited on the glassy carbon substrate at −1.00 V (**a**) and −0.25 V (**b**–**d**) for 60 s.

In the case of direct cathodic measurements, the quality of the *ex situ* bismuth film prepared from a plating solution containing 0.02 mol dm^−3^ Bi(NO_3_)_3_, 1 mol dm^−3^ HCl and 0.5 mol dm^−3^ KBr [7] determines its applicability. To gain a deeper insight into the morphology of the electrodeposited bismuth, scanning electron microscopic measurements were performed. Figure 2 shows the micrographs of bismuth-films obtained at two different plating potentials: −1.00 V (a) and at −0.25 V (b–d), and under the same experimental conditions (deposition time, plating solution, and the same glassy carbon substrate). As we have observed previously, such bismuth-films are characterized by the different morphologies [7,8].

As can be seen from the micrograph shown in Figure 2a, the film deposited at −1.00 V is a relatively compact layer that covers the whole surface. The film is non-adhesive, mechanically unstable, often with poor contact to the substrate. Due to the highly acidic nature of the plating solution, the bismuth electrodeposition is accompanied by hydrogen evolution, causing additional weakening of the bond between substrate electrode and bismuth layers, generating thus holes in the bismuth layer. Such films are often prone to falling off from the surface in the form of black compact flakes. With such kind of film on the glassy carbon surface, the residual current of the electrode exceeds 100 μA, which is unfavorable for the analytical measurements. 

In contrast to the film obtained at −1.00 V, the bismuth particles electrodeposited at −0.25 V (Figure 2b–d) cover only about one quarter of the surface area of the glassy carbon substrate. After the electrodeposition of the film, the electrode is to be treated appropriately, which involves its washing in 1.0 mol dm^−3^ HCl and then activation by potential cycling in the supporting electrolyte used for the measurement. Such treatment yields structural rearrangement [8] and, as it can be seen (micrograph b), the bismuth forms islands with average diameters between 14 and 40 μm [7]. The micrographs c and d depict the same surface at a higher magnification, to illustrate a remarkable crystalline structure in detail. As can be seen, the bismuth layer is formed of rhombohedral crystals resembling twigs evenly covering the entire surface of the glassy carbon substrate. Further, it can be observed that bismuth islands consist of thin fibers (c) densely and randomly arranged around the nucleation centers. In addition to bismuth nanofibers, making up the major part of the structure, it is also possible to notice nanorods with conical endings (d). Hence, such a surface is a hybrid of the carbon substrate and bismuth. This type of electrode has a gray reflection and the film is bound to the glassy carbon surface by adhesion forces that are stronger than in the previous case (deposition at −1.00 V).

**Scheme 1:**
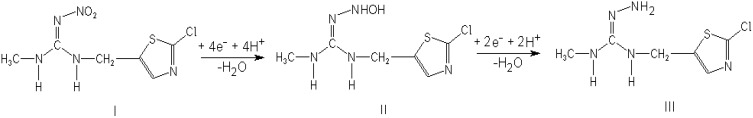
Proposed mechanism of clothianidin reduction at a mercury electrode [33].

It is well known that nitroguanidne neonicotinoids, e.g., imidacloprid, thiamethoxam and clothianidin, have electroactive functional groups reducible in a fairly negative potential range [7,8,9,28,29,30,31,32,33,34,35]. Relying on previous polarographic investigations, the determination of nitroguanidine neonicotinoids is based on the irreversible reduction of their electroactive nitro group to hydroxylamine and then to the corresponding amine [29,33,34]. The reduction process is complex, and, depending on the experimental conditions, one or more waves can be observed in a fairly negative potential range. The reduction mechanism of clothianidin (I) to hydroxylamine (II) and amine (III) on a mercury electrode is presented in Scheme 1 [33]. In the case of direct cathodic determination of imidacloprid and thiamethoxam on BiF-GCE, because of the narrower potential window, it is only possible to observe the first reduction step. Analogous signals are also observed for clothianidin.

**Figure 3:**
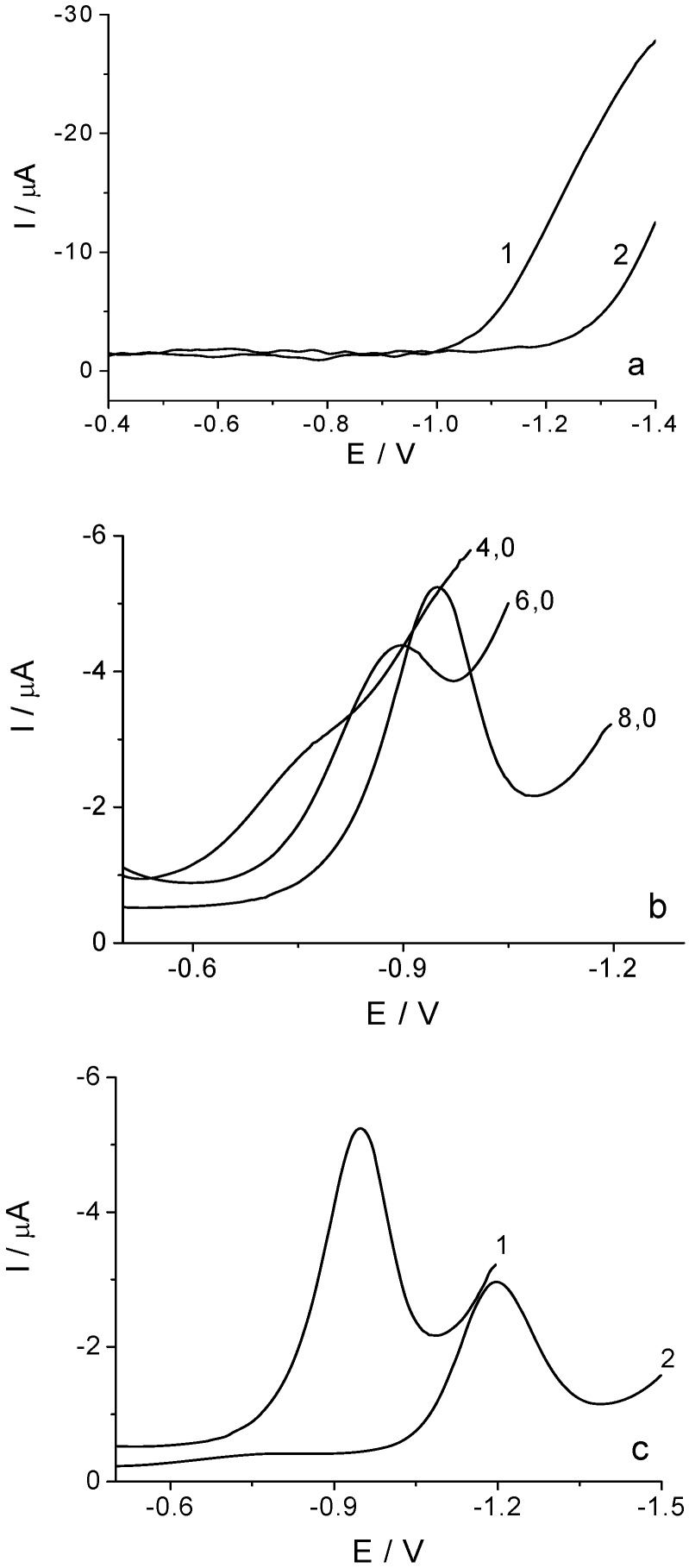
DPV curves at BiF-GCE recorded for the Britton-Robinson blank solutions (**a**) at pH 6.0 (1) and pH 8.0 (2). Clothianidin reduction peak at the pH values indicated on the curves (**b**). Comparison of DPV curves obtained under the same experimental conditions for the clothianidin reduction (**c**) at BiF-GCE (1) and GCE (2) at pH 8.0. Experimental parameters: *v* = 25 mV s^−1^, pulse amplitude = 50 mV, pulse width = 50 ms (**a**-**c**); c = 27.05 μg cm^−3^ (**b**, **c**).

Hence, a key prerequisite for the development of an analytical method for the determination of clothianidin is the preparation of a film electrode which will have a very low residual current just in this negative region of potential. The potential window of the BiF-GCE depends on the amount of electrodeposited bismuth. The increase in the amount of bismuth leads to the increase in the residual current [7] and decrease in the potential window (not shown). Besides, the potential window depends on the pH value of the medium. This can be seen from the DPV curves shown in Figure 3a, obtained for the same BiF-GCE at pH 6.0 (curve 1) and pH 8.0 (curve 2) of Britton-Robinson buffer solution, the representative blank solutions for the measurements. In the case of alkaline solution (curve 2) the potential of hydrogen evolution is shifted by about 300 mV to the negative direction compared to that for the pH 6.0 solution (curve 1), which, via the residual current level, influences the sensitivity of the determination.

The clothianidin reduction peak on the BiF-GCE is observed at potentials that are more negative than −0.5 V. Figure 3b illustrates how the signal characteristics, such as reduction potential, shape, and intensity, depend on the pH of the supporting electrolyte. Such a behavior can be explained by the significant role of protons in the complex reduction mechanism, in which the first step involves the reduction of the nitro group to hydroxylamine [29,33,34,35]. The DPV curves obtained for clothianidin show that in the media between pH 3.0 and 5.0 there is a reduction peak which overlaps with the hydrogen evolution signal. At pH 6.0, the reduction peak is separated significantly from the hydrogen evolution signal, and the well shaped and most intensive signal is being obtained at pH 8.0, so this pH was chosen for the analytical determination of clothianidin.

Comparative DPVs for clothianidin obtained under optimized analytical conditions on BiF-GCE (curve 1) and GCE (curve 2) are presented in Figure 3c. As can be seen, the reduction peaks at both electrodes are well defined, the peak potential is shifted to the positive direction by about 300 mV for BiFE, and the peak current obtained at this electrode is about two times higher than that obtained using GCE. A great advantage of the BiF-GCE over the GCE is that it is not sensitive to the presence of dissolved oxygen.

**Figure 4:**
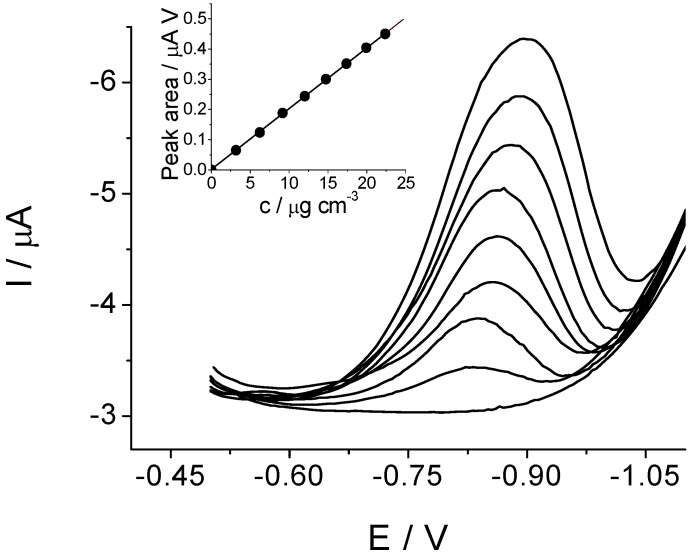
Differential pulse voltammograms recorded at the BiF-GCE for different concentrations of clothianidin in Britton–Robinson buffer solution pH 8.0. The inset shows the corresponding calibration plot.

The quantitative DPV determination of clothianidin was based on the linear relationship between the area of the peaks and concentrations (Figure 4). The linearity of the response was obtained in the range of 2.5−23 μg cm^−3^, while the limit of detection was 0.75 μg cm^−3^, and the limit of quantitation 2.5 μg cm^−3^.

**Figure 5:**
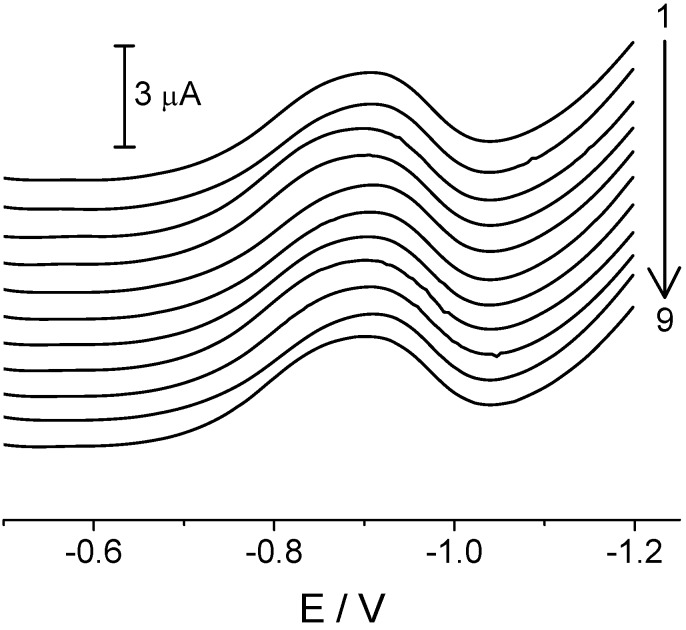
Reproducibility of the analytical signal for 22.4 μg cm^−3^ of clothianidin at BiF-GCE in 30 min time interval. Other experimental parameters are the same as in Figure 3.

Studies of the reproducibility of the BiF-GCE signal in the determination of clothianidin were also performed, first of all to check the signal stability and possible changes in its shape due to potential adsorption processes. As can be seen from Figure 5, the repeated measurements of a 22.4 μg cm^−3^ clothianidin solution show good reproducibility of the analytical signal over a time interval of approximately 30 min, with no significant changes in the electrode properties during the measurement. The relative standard deviation (RSD) was no higher than 1.5%.

Generally, the film deposited on the glassy carbon substrate modifies substantially the surface properties, showing improved sensitivity and reproducibility of the measurement. It is important to note that, similarly to the previously investigated neonicotinoids, the reduction signal of clothianidin appears at a potential that is by about 300 mV less negative than in the case of the bare GCE. Although BiFEs have many advantages, their potential window, apart from the pH, electrochemical pretreatment, *etc.*, is limited by the nature of the bismuth deposit, as well as by the quality of the film. Furthermore, our film type is moderately sensitive to the mechanical damage, but this is a common weakness of *ex situ* plated thin-film electrodes.

### 2.2. Bulk Modified Tricresyl Phosphate-Based Carbon Paste Electrode in the Imidacloprid Analysis

Another approach yielding a bulk modified material involves the introduction of a third component into the carbon paste. Such a modification of the CPE is highly desirable because of the expectation that an electrode modified with third component would exhibit efficient catalysis, high stability, and comparatively simple renewability. Recently, a new type of bismuth modified electrode was introduced, containing bismuth powder directly admixed in carbon paste [16,17,44]. It is reported that it combines the advantages of bismuth electrodes and carbon paste electrodes. Our previous experiments showed that the tricresyl phosphate is a convenient binding liquid because imparts polar behavior to the carbon paste, which allows sensitive determination of selected neonicotinoid insecticides [30,31,32]. The objective of this part of investigation was to compare the behavior of the unmodified TCP-CPE with the modified one in the determination of imidacloprid. 

**Figure 6:**
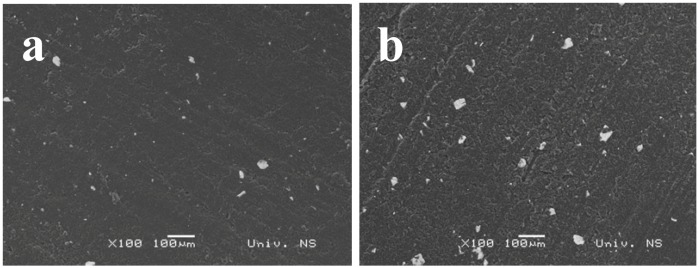
Scanning electron micrographs of Bi5-TCP-CPE (**a**) and Bi20-TCP-CPE (**b**) taken at a magnification of 100×.

Figure 6 shows the morphology of the TCP-CPE modified with 5 (Bi5-TCP-CPE, a) and 20 (Bi20-TCP-CPE, b) w∕w% of Bi powder. It can be seen that the basic carbon matrix contains a homogenous solid phase and randomly distributed inhomogeneities which are more frequent in the case of the TCP-CPE modified with 20% Bi powder.

**Figure 7:**
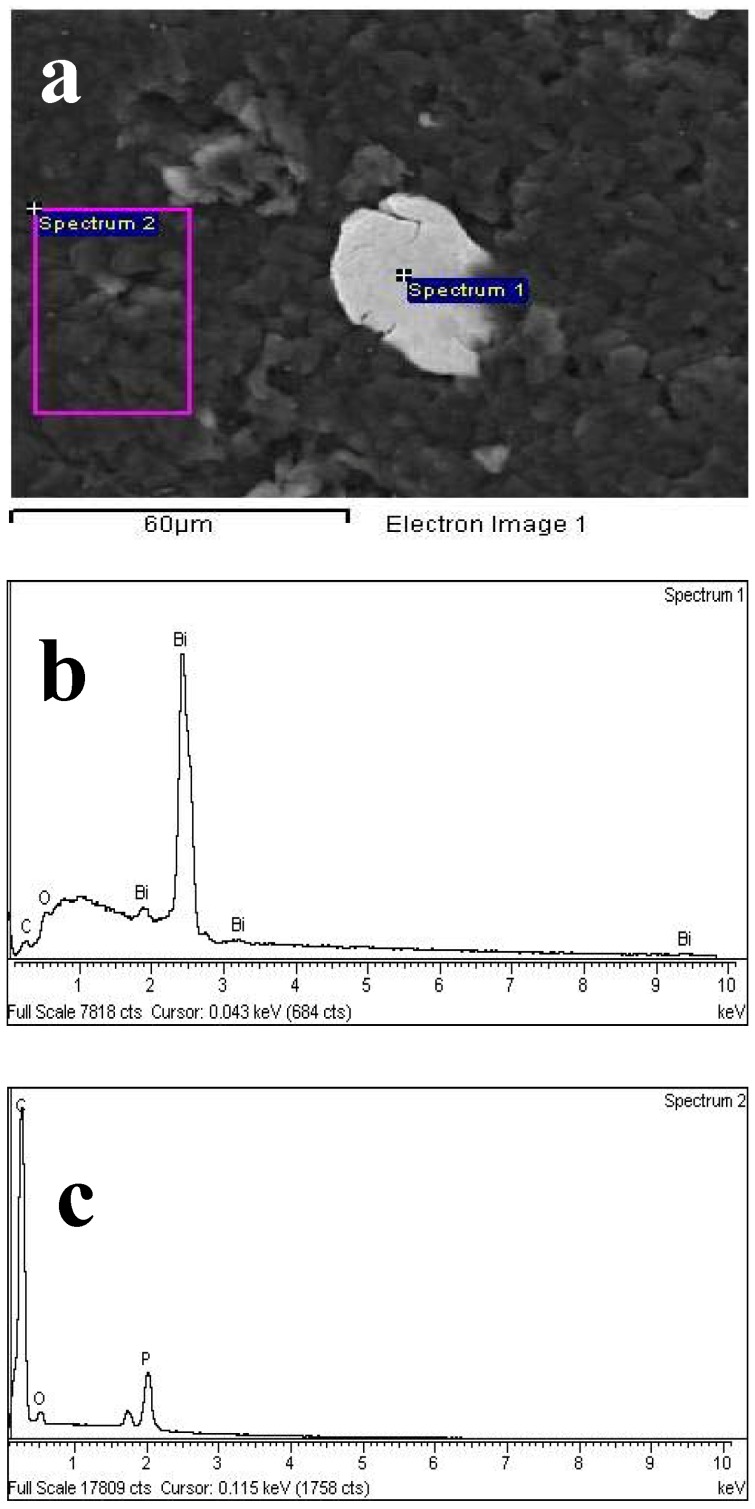
Representative part of the Bi5-TCP-CPE surface (**a**). EDS microanalysis spectra of the bismuth island surface area (**b**) and of the carbon paste matrix (**c**).

The energy dispersive spectrometric (EDS) measurements (Figure 7) performed on two different parts of the representative Bi5-TCP-CPE surface (a), selected so that one of them contains the inhomogeneity, proved that the inhomogeneities are in fact bismuth islands (spectrum 1, b) in the tricresyl phosphate-based carbon paste matrix (spectrum 2, c).

**Scheme 2:**
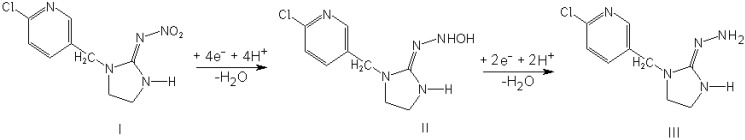
Proposed mechanism of imidacloprid reduction at a mercury electrode [29,34].

As was mentioned earlier, imidacloprid (I) at mercury electrode is reduced to hydroxylamine (II) and amine (III) derivatives (Scheme 2). Our previous experience [8,28,30,31,32] showed that on the carbon-based working electrodes only the first reduction step can be observed because of the narrower potential window, like in the case of mercury. This behavior was observed in the case of both unmodified and modified TCP-CPEs.

**Figure 8:**
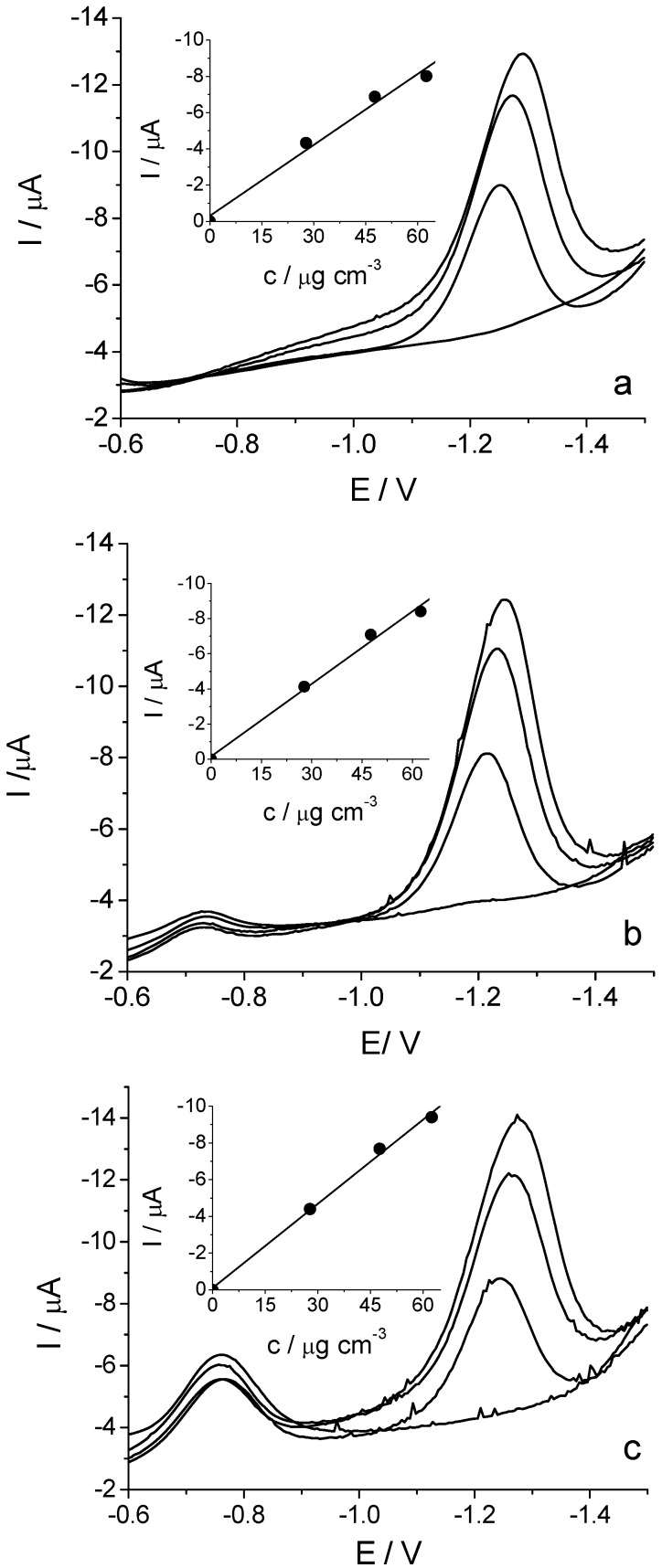
Differential pulse voltammograms recorded at TCP-CPE (**a**), Bi5-TCP-CPE (**b**) and Bi20-TCP-CPE (**c**) for the different concentrations of imidacloprid in Britton–Robinson buffer solution pH 8.0.

The main drawback of carbon paste electrodes is the presence of the oxygen peak on the DPV baseline obtained with untreated TCP-CPE (not shown). Purging of the solution with nitrogen in combination with electrochemical pretreatment of the electrode stabilizes the baseline and allows the measurements in a fairly negative potential range. However, the bismuth modified TCP-CPE does not require purging with an inert gas. The applicability of the unmodified and bulk modified TCP-CPE as working electrodes for the determination of imidacloprid was compared under the same experimental conditions (Figure 8). In all cases, the reduction peak is observed at around −1.2 V, but the reduction signals are different. In comparison with TCP-CPE (a), a potential shift by about 50 mV towards the less negative potentials can be observed when using bismuth modified electrodes (b, c). It should be mentioned that in the case of bulk modified electrodes the DPV curves have another smaller reduction peak at about −0.75 V. This signal increases as the bismuth content increases, but it does not affect the quality of the imidacloprid reduction signal. Further studies are needed to elucidate the origin of this peak. The increase in the bismuth content resulted in similar peak currents for the same imidacloprid concentration, but the noise, especially of the spike type, increased (c). This phenomenon may have a negative consequence for the sensitivity of the determination of imidacloprid, especially at the lower concentrations. However, the main advantage of bismuth modified TCP-CPE is its lower sensitivity to the presence of oxygen, so that there is no need to purge the solution with nitrogen. Bearing in mind the intensive noise effect obseved in the case of Bi20-TCP-CPE, this electrode was not considered further.

The estimated values for the limit of quantification were similar for TCP-CPE and Bi5-TCP-CPE, reaching 1.7 µg cm^−3^ and 1.6 µg cm^−3^, respectively. Reproducibility studies were performed, first of all to check the signal stability and possible changes in the signal shape. Six repeated measurements of the 7.73 µg cm^−3^ imidacloprid solution showed good reproducibility of the analytical signal in the time interval of approximately 30 min for TCP-CPE and Bi5-TCP-CPE, with no significant changes in the electrode properties during the measurements. The RSD was not higher than 2.4%.

If high accuracy of measurement is to be achieved, it is recommended to repeat the calibration procedure after each surface renewal and new electrode preparation (especially in the case of bulk modified CPEs). As it has already been observed [32], the TCP-CPEs can be used for a shorter time (several days) than the most widely used SO-based CPEs, mostly due to the volatility of the TCP binder. Enveloping of the TCP-CPE with Parafilm^®^ extended its lifetime, and it remained usable for several months [32].

Further experiments are needed to confirm the applicability of the bismuth modified TCP-CPE for the analysis of real samples. As it was shown for the unmodified TCP-CPE [30,31,32], which was used for the determination of imidacloprid and thiamethoxam in different commercial formulations and river water samples, the bismuth modified TCP-CPE could have a wide applicability, especially because of the lower sensitivity for the presence of the dissolved oxygen.

Taking into account all the above, we can conclude that the DPV using an *ex situ* bismuth-film modified GCE and carbon paste electrode bulk-modified with bismuth can be a fast and inexpensive tool for the detection and determination of selected nitroguanidine neonicotinoids in the presence of the dissolved oxygen. Further improvement of the sensitivity of the metod can be achieved by the preconcentration of the insecticide sample. Furthermore, both electrode types can serve as electrochemical detectors in flow injection, sequential injection, or chromatographic arrangements. 

Bearing in mind that the highly toxic mercury film electrode very often can be replaced with electrodes surface or bulk modified with bismuth, the exploratory research in this area can give environmentally-friendly, simple and cheap working electrodes for the determination of different electroactive analytes.

## 3. Experimental

### 3.1. Chemicals and Solutions

All chemicals used were of analytical reagent grade and the solutions were prepared in doubly distilled water. Analytical standards of imidacloprid and clothianidin (Sigma-Aldrich Laborchemikalien GmbH, Germany) were of 99.9% purity. The concentration of the imidacloprid and clothianidin stock solutions were 166.7 and 162.3 μg cm^−3^, and they were further diluted as required. Britton-Robinson buffer solutions for voltammetric characterization and determination were prepared from a stock solution containing 0.04 M phosphoric (Merck, Germany), boric (Merck) and acetic (Merck) acids, by adding 0.2 M sodium hydroxide (Merck) to obtain the required pH value. Bi(NO_3_)_3_ × 5H_2_O (extra pure) was purchased from Riedel-de Haën, Switzerland. Carbon pastes were made from CR 5 graphite powder (Maziva Týn, Czech Republic) and tricresyl phosphate (mixture of isomers, Sigma-Aldrich Chemie GmbH, Switzerland) as a pasting liquid, with or without addition of bismuth powder (99.99+%, 100 mesh, Aldrich Chem. Co., USA). 

### 3.2. Apparatus

Voltammetric experiments were performed on an Autolab electrochemical analyzer (PGSTAT12, Ecochemie, The Netherlands) operated via GPES 4.9 software (Ecochemie). The cell stand included a three-electrode system with TCP-CPE / Bi-TCP-CPE or GCE / BiF-GCE (Amel, Italy), as working, an SCE (Amel) as reference, and a platinum (Amel) auxiliary electrode. The diameter of the CPEs and GCEs was 2 and 3 mm, respectively. All potentials are quoted *vs.* SCE reference electrode.

The surface morphology of BiF-GCEs and Bi-TCP-CPEs was studied on a Jeol JSM-6460LV scanning electron microscope (Japan Electron Optics Laboratory, Japan). The EDS microanalysis of Bi5-TCP-CPE was performed on an INCA microanalysis system (Oxford Instruments, United Kingdom).

All pH measurements were made with the aid of a digital pH meter using a combined glass electrode.

### 3.3. Procedures

#### 3.3.1. Bismuth-Film Deposition

Two different plating potentials were used: −1.00 V and −0.25 V. The procedure was carried out *ex situ* in a solution consisting of 0.02 mol dm^−3^ Bi(NO_3_)_3_, 1 mol dm^−3^ HCl and 0.5 mol dm^−3^ KBr, for 60 s [7], without stirring. Following this, the BiF-GCEs were rinsed lightly with 1 mol dm^−3^ HCl. Before the film deposition, the polished GCE was treated by *in situ* potential cycling (10 cycles) in the plating solution from 0.00 V to −0.60 V. After that, a potential of +0.20 V was applied for about 30 s to clean the surface. Such treated electrode was ready for the film preparation. To remove the film, a potential of +0.20 V was applied.

The unmodified TCP-CPE was made by hand-mixing of graphite powder (0.25 g) with tricresyl phosphate (0.1 cm^3^) in a porcelain mortar. The modified pastes (Bi5-TCP-CPE and Bi20-TCP-CPE) were prepared by replacing 5% or 20% (w/w) of the carbon powder with bismuth powder.

#### 3.3.2. Voltammetry

Clothianidin and imidacloprid were measured in 5.00 cm^3^ of the solution of different concentration, to which 5.00 cm^3^ of Britton-Robinson buffer solution was added. The solution was purged with high-purity nitrogen for 6 min only in the case of the measurement with TCP-CPE. All data were obtained at ambient temperature.

#### 3.3.3. Scanning Electron Microscopy

The electrodeposited bismuth-films and the bulk-modified carbon paste electrodes were examined on a Jeol scanning electron microscope with an accelerating voltage of 20 kV in low vacuum mode, at the working distance of 20 mm, using a secondary electron detector. Prior to the SEM examination, the electrode plated with bismuth at −0.25 V was rinsed slightly with 1.0 mol dm^−3^ HCl, conditioned by potential cycling in Britton-Robinson buffer solution (pH 8.0), dried and observed. Because of the mechanical instability of the film deposited on GCE at −1.00 V it was only dried before observation. The EDS microanalyses were carried out on a representative surface area. The bulk modified Bi-TCP-CPEs were directly measured without any surface pretreatment.

## 4. Conclusions

The BiF-GCE, a non-toxic electrode, was applied for direct cathodic voltammetric determination of the insecticide clothianidin. The determination was performed by DPV in Britton-Robinson buffer solution pH 8.0. The voltammetric response was linear in the range of 2.5–23 μg cm^−3^, while the limit of detection was 0.75 μg cm^−3^, and the limit of quantitation 2.5 μg cm^−3^. The relative standard deviation was below 1.5%.

Analytical performances of tricresyl phosphate-based carbon paste electrodes bulk modified with two amounts of bismuth (5 and 20 w/w%) were compared with that of the bare TCP-CPE for the determination of imidacloprid. Although the TCP-CPE and bismuth-modified TCP-CPEs have similar analytical performances, the electrode with 5% of Bi is advantageous as it can be also used in the presence of dissolved oxygen in the analyzed solution and the obtained DPV curves are devoid of randomly distributed spiky noise signals which appear with the electrodes containing 20% Bi.
